# Bibliometric analysis and visualization of quorum sensing research over the last two decade

**DOI:** 10.3389/fmicb.2024.1366760

**Published:** 2024-04-05

**Authors:** Xinghan Chen, Jiaqi Li, Ruohan Liao, Xiujun Shi, Yan Xing, Xuewen Xu, Haitao Xiao, Dongqin Xiao

**Affiliations:** ^1^Research Institute of Tissue Engineering and Stem Cells, Nanchong Central Hospital, The Second Clinical College of North Sichuan Medical College, Nanchong, Sichuan, China; ^2^Department of Burns and Plastic Surgery, West China Hospital, Sichuan University, Chengdu, Sichuan, China; ^3^Department of Dermatology, West China Hospital, Sichuan University, Chengdu, Sichuan, China

**Keywords:** quorum sensing, research trends, bibliometric analysis, biclustering analysis, visualization

## Abstract

**Background:**

Quorum sensing (QS) research stands as a pivotal and multifaceted domain within microbiology, holding profound implications across various scientific disciplines. This bibliometric analysis seeks to offer an extensive overview of QS research, covering the period from 2004 to 2023. It aims to elucidate the hotspots, trends, and the evolving dynamics within this research domain.

**Methods:**

We conducted an exhaustive review of the literature, employing meticulous data curation from the Science Citation Index Extension (SCI-E) within the Web of Science (WOS) database. Subsequently, our survey delves into evolving publication trends, the constellation of influential authors and institutions, key journals shaping the discourse, global collaborative networks, and thematic hotspots that define the QS research field.

**Results:**

The findings demonstrate a consistent and growing interest in QS research throughout the years, encompassing a substantial dataset of 4,849 analyzed articles. Journals such as Frontiers in Microbiology have emerged as significant contributor to the QS literature, highlighting the increasing recognition of QS's importance across various research fields. Influential research in the realm of QS often centers on microbial communication, biofilm formation, and the development of QS inhibitors. Notably, leading countries engaged in QS research include the United States, China, and India. Moreover, the analysis identifies research focal points spanning diverse domains, including pharmacological properties, genetics and metabolic pathways, as well as physiological and signal transduction mechanisms, reaffirming the multidisciplinary character of QS research.

**Conclusion:**

This bibliometric exploration provides a panoramic overview of the current state of QS research. The data portrays a consistent trend of expansion and advancement within this domain, signaling numerous prospects for forthcoming research and development. Scholars and stakeholders engaged in the QS field can harness these findings to navigate the evolving terrain with precision and speed, thereby enhancing our comprehension and utilization of QS in various scientific and clinical domains.

## Introduction

Quorum sensing (QS) is a captivating and essential cellular communication process that transcends species boundaries, involving the exchange of signaling molecules among diverse microbial communities (Grandclément et al., 2016; Prescott and Decho, 2020). It has emerged as a pivotal area of research since its discovery in the marine luminous bacterium *V. fischeri* in the 1970's (Arnold et al., 1972). This groundbreaking finding has propelled QS into a wide array of applications, spanning from medical therapeutics targeting bacterial virulence to environmental strategies for pollutant degradation. This phenomenon serves as the cornerstone of intricate intercellular dialogues that underpin collective behaviors within bacterial populations (Eickhoff and Bassler, 2018; Subramani and Jayaprakashvel, 2019; Wu et al., 2022). At its core, QS enables bacteria to gauge their population density and respond collectively to changing environmental conditions (Cornforth et al., 2014; Popat et al., 2015). This dynamic process has far-reaching implications across a spectrum of fields, from the formation of biofilms to the development of QS inhibitors.

Biofilms, structured communities of bacteria embedded in self-produced extracellular matrices, represent a paradigmatic outcome of QS in action (Irie and Parsek, 2008; Steinberg and Kolodkin-Gal, 2015). Bacteria coordinate their activities within biofilms, enhancing their resistance to external stresses, antibiotics, and immune responses (Fisher et al., 2017; Sharma et al., 2019). Understanding the mechanisms underlying biofilm formation is a central focus of QS research (Li et al., 2022), as it carries significant implications for infection control. Furthermore, the development of QS inhibitors has emerged as a promising strategy to disrupt bacterial communication and attenuate the virulence of pathogenic species (Tay and Yew, 2013; Scutera et al., 2014).

In the realm of practical applications, QS research spans across diverse domains, including medicine, agriculture, food science, and sewage treatment (Annous et al., 2009; Sharma and Jangid, 2014; Huang et al., 2016). In medicine, it has profound implications for combating bacterial infections, particularly those involving multidrug-resistant strains (Kiran et al., 2011; Alibi et al., 2020). Agriculture benefits from insights into plant-microbe interactions and the potential for environmentally friendly microorganism management (Berg, 2009). In food science, QS impacts food safety and preservation (Skandamis and Nychas, 2012; Li et al., 2018). Moreover, QS research has transcended disciplinary boundaries, in combination with materials science, antimicrobial coatings have been developed that possess the ability to regulate and inhibit QS, enhancing the efficiency of wastewater treatment facilities (Shrout and Nerenberg, 2012; Siddiqui et al., 2015). And QS research has transcended disciplinary boundaries, for instance, QS-triggered microbial fuel cells have showed significant application prospects in electricity generation and wastewater treatment (Monzon et al., 2016; Sivasankar et al., 2019). This multidisciplinary approach underscores the far-reaching impact of QS research and its potential to address an array of challenges across diverse fields.

Bibliometrics serves as a quantitative approach utilized to analyze and assess scientific literature by employing statistical techniques (Kim et al., 2020). This methodology is instrumental in identifying research focal points and emerging trends within a given field, offering valuable insights into its current status and providing direction for future research initiatives. As an illustration, researchers have effectively employed bibliometric analysis to explore the evolving research landscape concerning subjects like microneedles, chronic wounds and transdermal drug delivery (Chen et al., 2022, 2023a,b). Providing an impartial portrayal of research trends, bibliometrics analysis plays a crucial role in guiding and molding the course of future research endeavors.

This investigation aims to comprehensively assess the existing body of literature concerning QS, utilizing a bibliometric analysis approach. The objective is to enhance understanding within the QS research domain by employing co-word biclustering analysis. The expected outcomes of this study are poised to assist researchers in achieving a more precise and efficient grasp of QS research trends and advancements.

## Methods

### Data source and search methods

**Figure 1:**
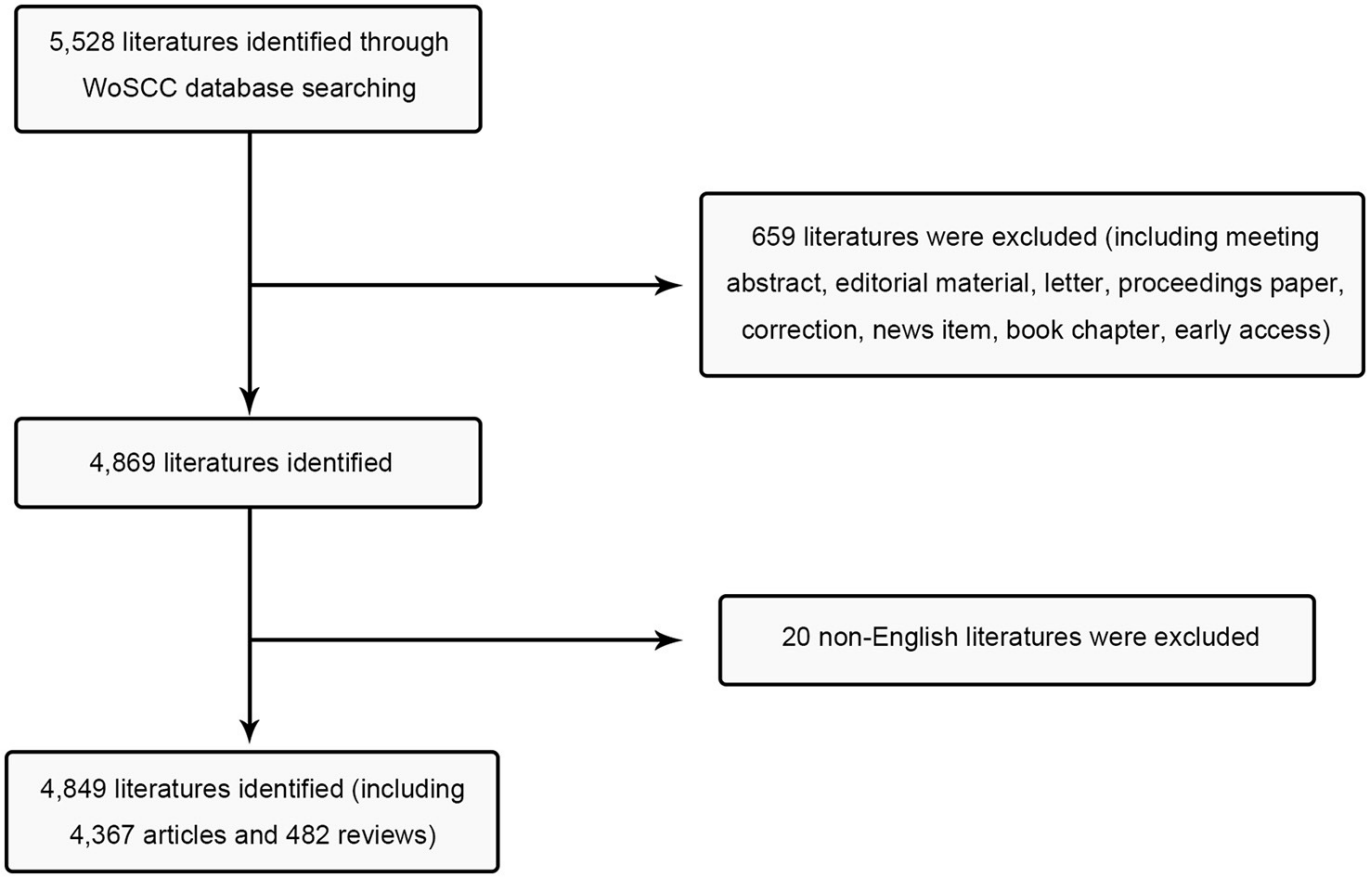
Flow chart of inclusion and exclusion.

We conducted a search on the Science Citation Index Extension (SCI-E) within the Web of Science (WOS) database, using the following search criteria: articles with “quorum sensing” in the title (TI) and written in English, covering the period from January 1, 2004, to December 31, 2023. A total of 5,528 articles were retrieved. Subsequently, we excluded specific types of literature, such as early access, book chapters, news items, corrections, proceedings papers, letters, editorial materials, and meeting abstracts, and used Photoshop V2020 to produce Figure 1. For co-word clustering analysis, we employed the retrieval function provided by the NCBI for Medical Subject Heading (MeSH) terms, with the analysis period set from 2004 to 2023 and “quorum sensing” designated as the MeSH term. All publications were retrieved as of December 21, 2023, to minimize potential biases stemming from database updates.

### Data collection

Two independent reviewers assessed the search outcomes, considering various factors including the H-index, citation count, author details, geographical location, publication date, and title. These reviewers achieved a substantial overall agreement ratio of 0.90. In cases where disparities in assessments arose, a third reviewer was consulted to make the final determination regarding data inclusion. The data used for analysis was imported into two separate platforms: an online Bibliometrics analysis platform (http://bibliometric.com/) and VOSviewer V1.6.17 (Leiden University, Leiden, the Netherlands), both using the “Tab Delimited File” format. For CiteSpace V 6.2.R4 (Drexel University, Philadelphia, PA, United States), the data was uploaded in the “Plain Text File” format. Furthermore, MeSH terms obtained from NCBI were initially formatted as “PubMed” and subsequently input into BICOMB V2.02 for analysis. Subsequently, gCLUTO V1.0 (Graphical Clustering Toolkit) was employed to visualize biclustering based on the co-word matrix file generated during the investigation.

## Data investigation

### Bibliometric analysis and geographical distribution

We utilized the Bibliometrics online analysis platform to visualize international collaborations and contributions at national/regional levels. VOSviewer was employed for creating a clear clustering visualization, with a primary focus on evaluating inter-agency cooperation among institutions. Additionally, we employed the number of journals published as the standard for density clustering visualization. Within VOSviewer, we selected the “bibliographic data-based map creation” data format and specified “Co-authorship” as the analysis category, resulting in the generation of the “Density Visualization” graphic. Furthermore, CiteSpace was utilized to forecast future research directions, identify research trends, and pinpoint emerging keywords over time. To ensure data accuracy, we recommended applying the “Remove duplicates” function before configuring keywords as node types, with “the number of states” set to 2. Journal impact factors (IF) were determined by referencing the 2023 Journal Citation Report (JCR).

### Co-word biclustering analysis

We utilized BICOMB and gCLUTO to perform biclustering analysis on significant MeSH terms and MeSH subheadings, with the objective of identifying key research focal points. In the BICOMB software, we formatted the data in the “PubMed” style and imported the relevant MeSH term data. By using the “Extract” function, we configured it to include both the main topic and sub-topic. Subsequently, specialized software was employed to convert the primary MeSH terms into a matrix, resulting in a co-word matrix highlighting frequently occurring MeSH terms. This co-word matrix was then input into gCLUTO, where we defined parameters, including the “Number of Clusters,” and selected the “Repeated Bisection” as the cluster method. Finally, matrix and mountain visualization techniques were applied to present the outcomes of the biclustering analysis, offering valuable insights into research hotspots within the QS field.

## Results

### Investigation of publications output

**Figure 2:**
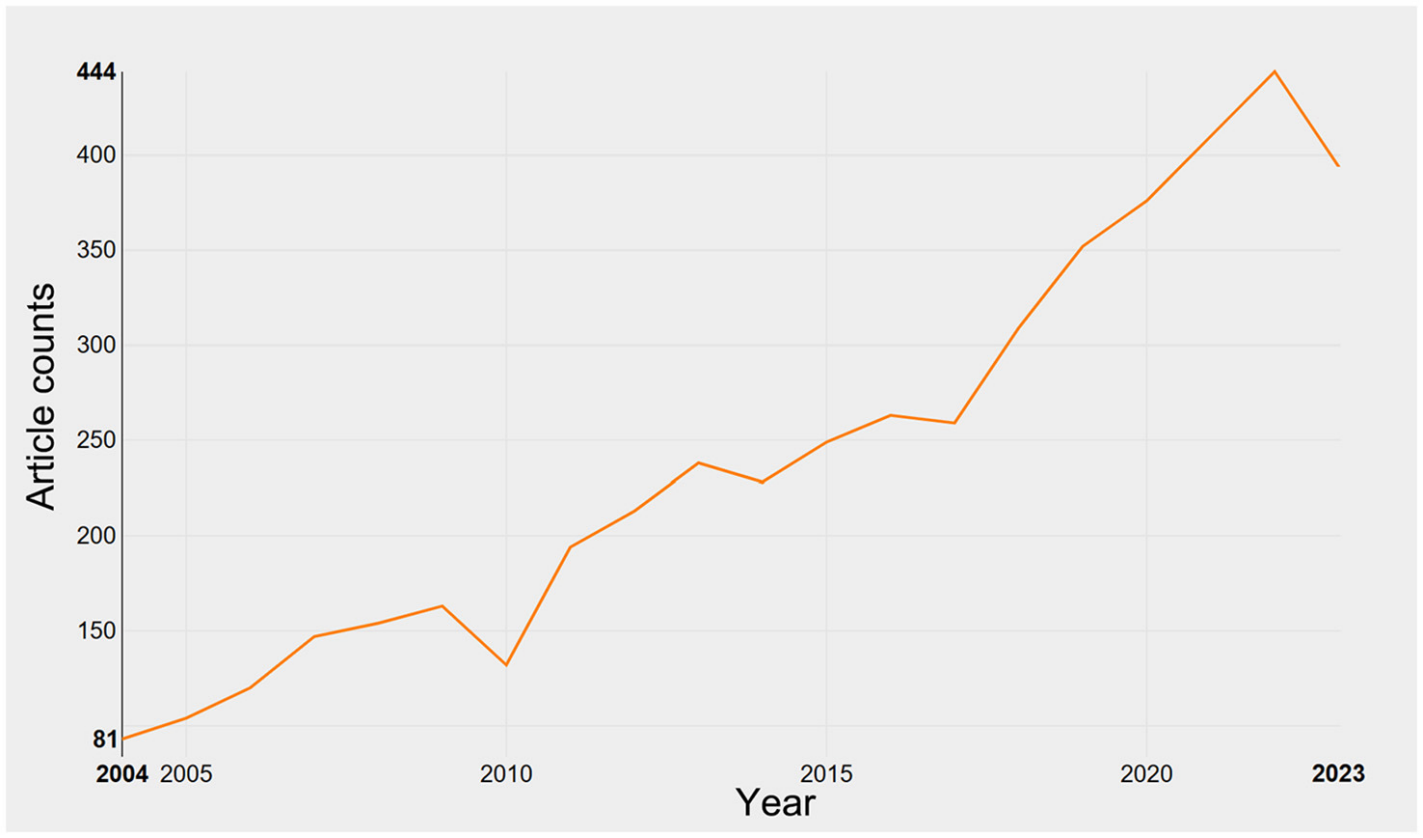
Growth of publications on quorum sensing (2004–2023).

As per our inclusion criteria, a combined total of 4,367 research articles and 482 review articles pertaining to QS were identified (Figure 1). An analysis of publication trends from 2004 to 2023 reveals a consistent growth pattern, with a notable upsurge in 2017 (Figure 2). It is worth noting that, as of the present date, the count of articles published in 2023 has reached 394, signifying nearly 4-fold increase compared to the number published in 2004. The lower number of publications in 2023 compared to 2022 could be attributed to the data collection not encompassing the entire year and potential delays in the online publication of some works.

### The contributions of nations and institutions to global publications

**Figure 3:**
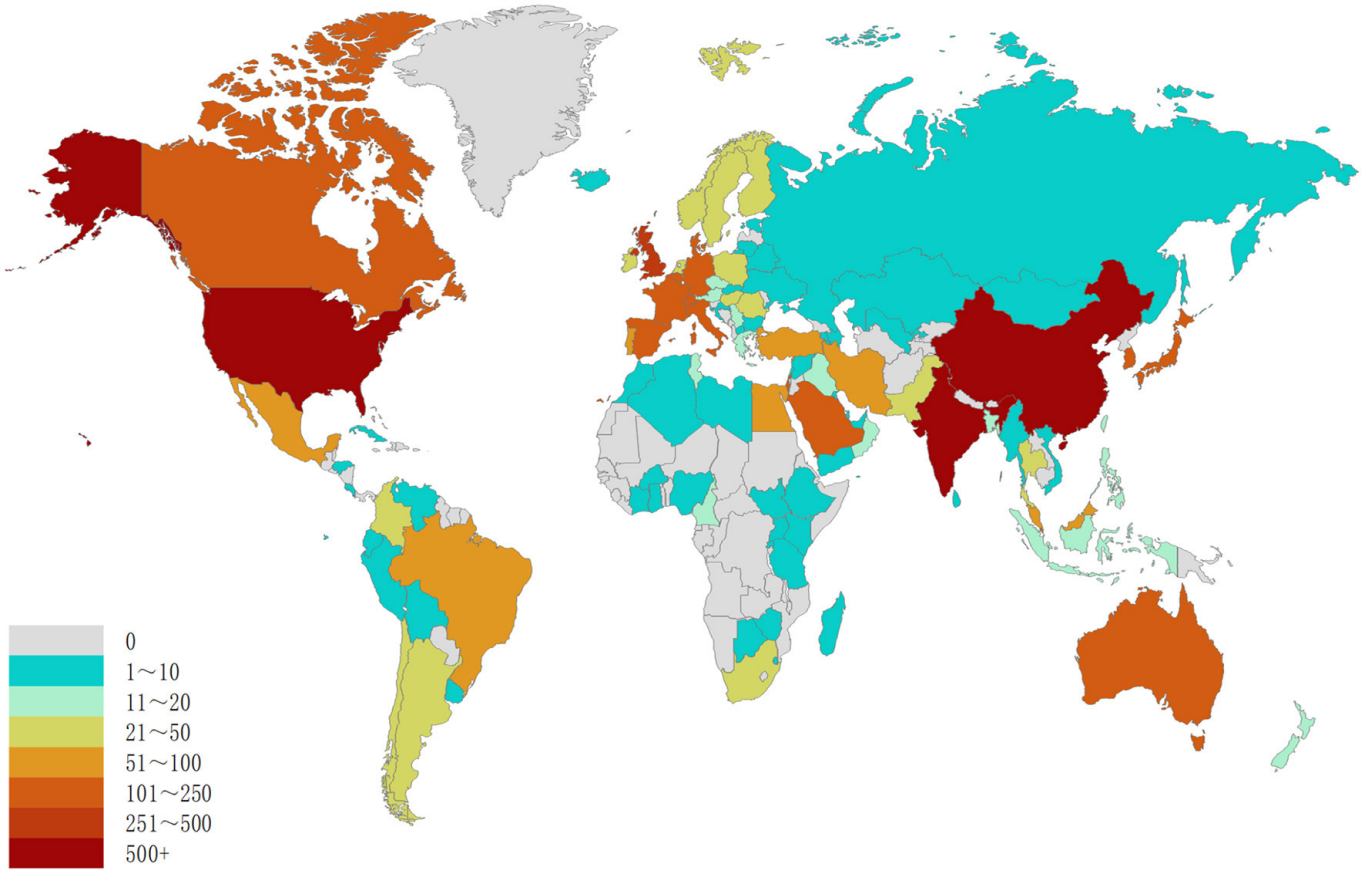
Geographical distribution of retrieved articles in quorum sensing (2004–2023).

**Figure 4:**
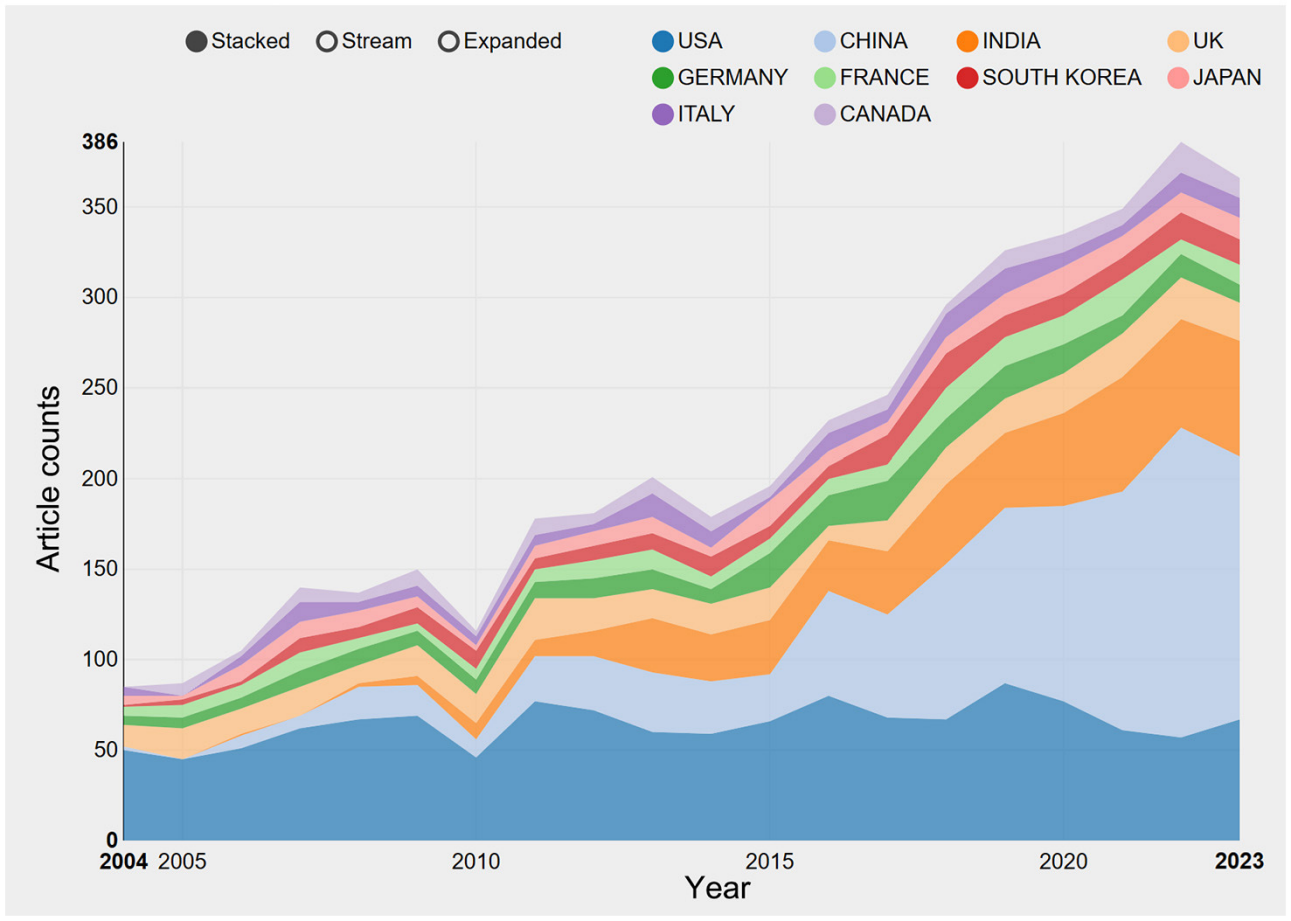
The growth trends of the top 10 nations/regions in quorum sensing (2004–2023).

**Figure 5:**
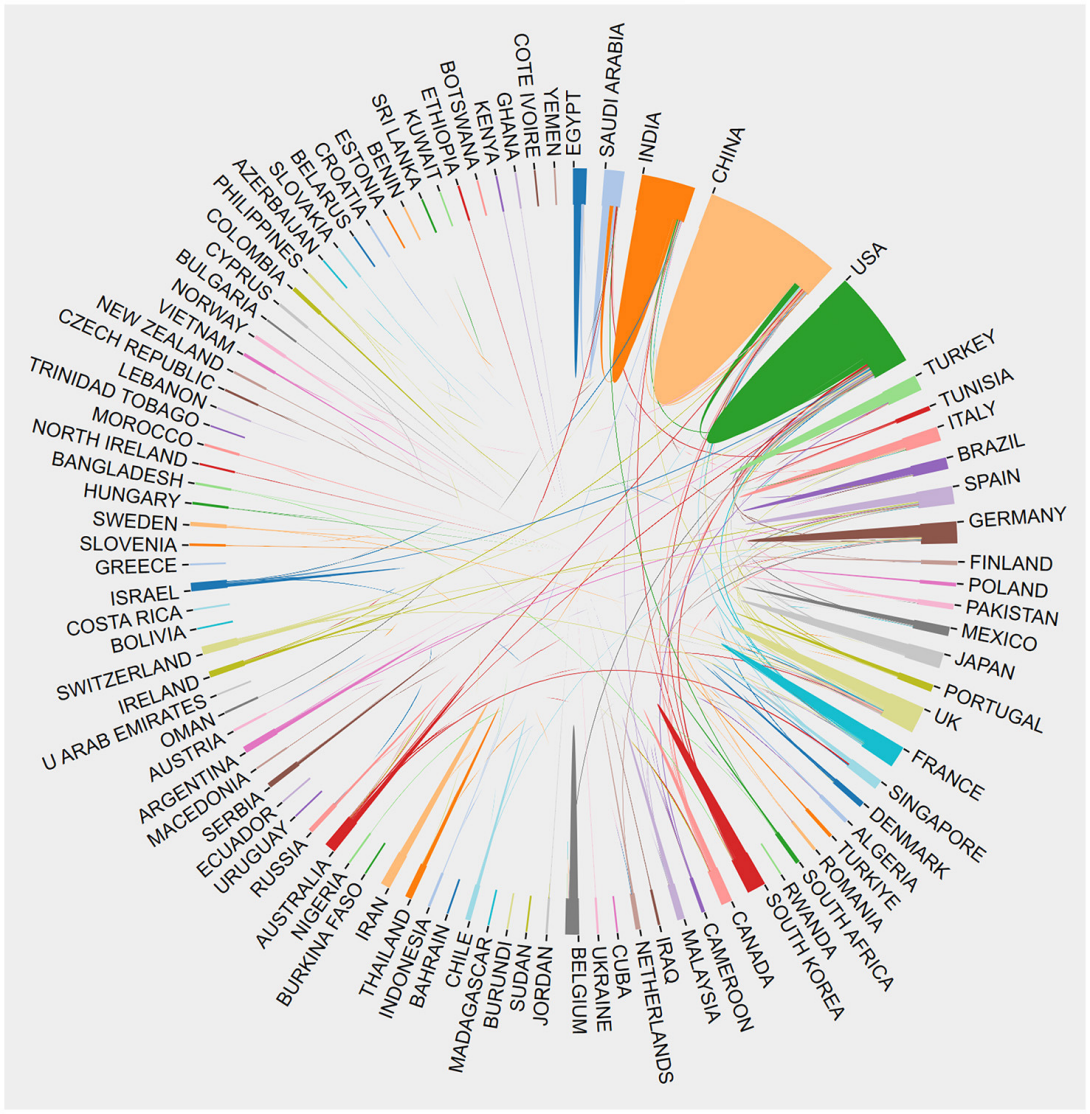
The network map of country/region' cooperation.

**Figure 6:**
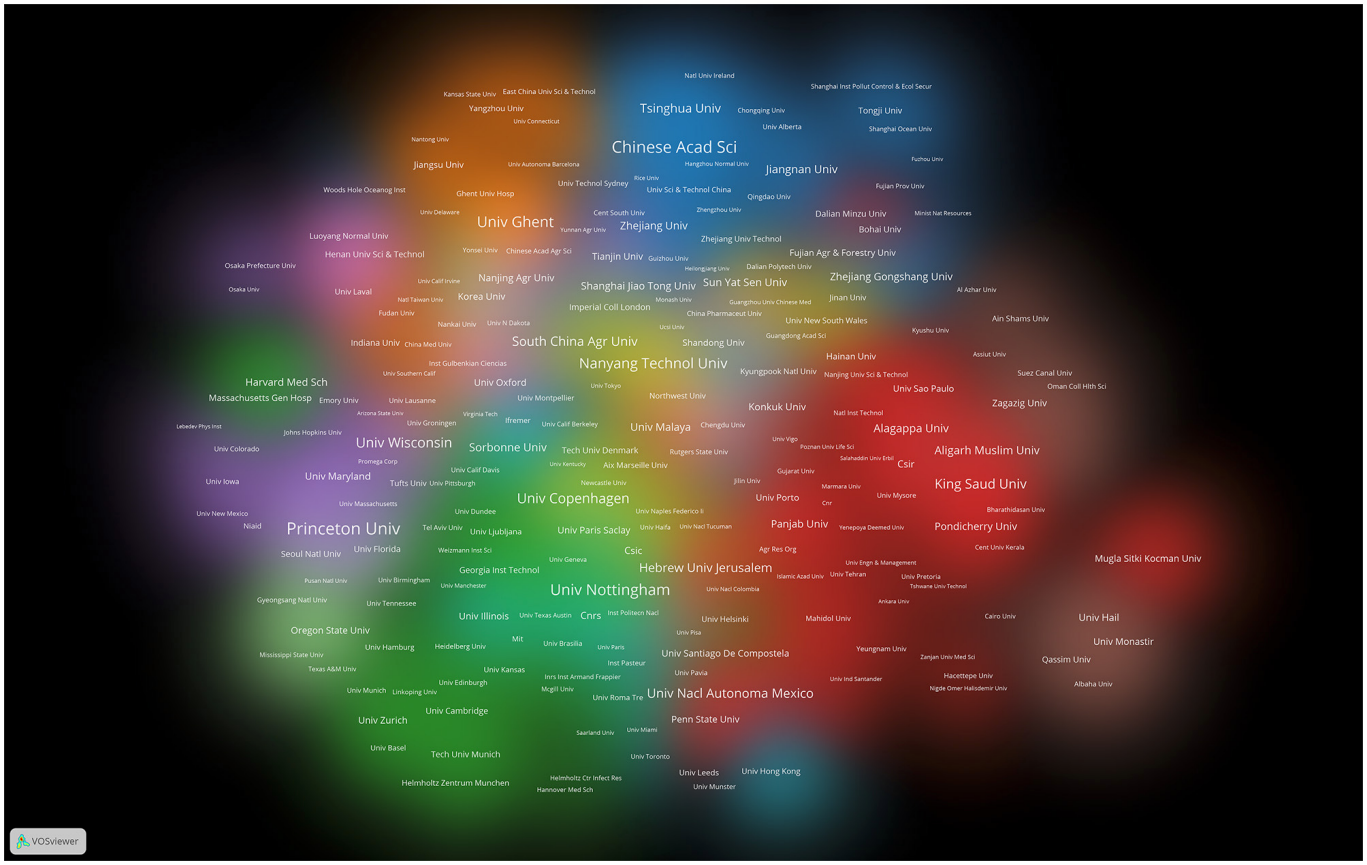
Cluster density visualization map of institutions on quorum sensing (2004–2023).

The exploration of QS spans across a diverse spectrum of 101 countries and regions. Upon data import, a thermal map (Figure 3) vividly illustrates that these articles predominantly concentrate in East Asia, North America, and Western Europe. Significantly, the United States (*n* = 1,292) emerges as the foremost contributor, followed by China (*n* = 1,048) and India (*n* = 514; Table 1). Figure 4 showcases the publication growth trajectories of major countries. An analysis of collaboration among countries and regions indicates that there is a close level of cooperation between the United States and China (Figure 5). Centrality serves as a pivotal indicator of a country's global collaboration involvement, with elevated centrality values indicating a more substantial impact. The results indicate that the United States exhibits the highest centrality (center = 0.51), followed closely by China (0.32) and England (0.25). Among research institutions, the top five comprise University of Nottingham (*n* = 129), Center National De La Recherche Scientifique (*n* = 124), Princeton University (*n* = 112), University of Wisconsin System (*n* = 90), and Howard Hughes Medical Institute (*n* = 89; Table 1). Employing VOSviewer, we scrutinized inter-agency collaborations through co-authorship and visualized these alliances as density clusters (Figure 6). This analysis of inter-agency partnerships reveals 10 distinct clusters, each represented by a unique color. Within the same color cluster, the level of collaboration among institutions is relatively high, exemplified by the joint publication of documents with shared affiliations and instances where a scholar holds concurrent positions across different institutions.

**Table 1:** Top 10 productive country/region and institutions on quorum sensing (2004–2023).

**Rank**	**Country/region**	**Article counts**	**Centrality**	**Total number of citations**	**Average number of citations**	**Institutions**	**Article counts**	**Total number of citations**
1	USA	1,292	0.51	77,508	59.99	University of Nottingham	129	9,895
2	China	1,048	0.32	22,044	21.03	Center National De La Recherche Scientifique	124	4,163
3	India	514	0.12	12,590	24.49	Princeton University	112	15,931
4	England	318	0.25	17,273	54.32	University of Wisconsin System	90	10,809
5	Germany	232	0.14	10,497	45.25	Howard Hughes Medical Institute	89	10,809
6	France	195	0.17	7,103	36.43	Chinese Academy of Sciences	88	2,424
7	South Korea	188	0.01	5,886	31.31	Ghent University	85	4,305
8	Japan	172	0.11	4,393	25.54	University of Washington	83	7,228
9	Italy	153	0.05	5,154	33.69	University of Copenhagen	81	6,284
10	Canada	151	0.09	7,072	46.83	Egyptian Knowledge Bank	80	1,363

### Journals publishing research on quorum sensing

**Figure 7:**
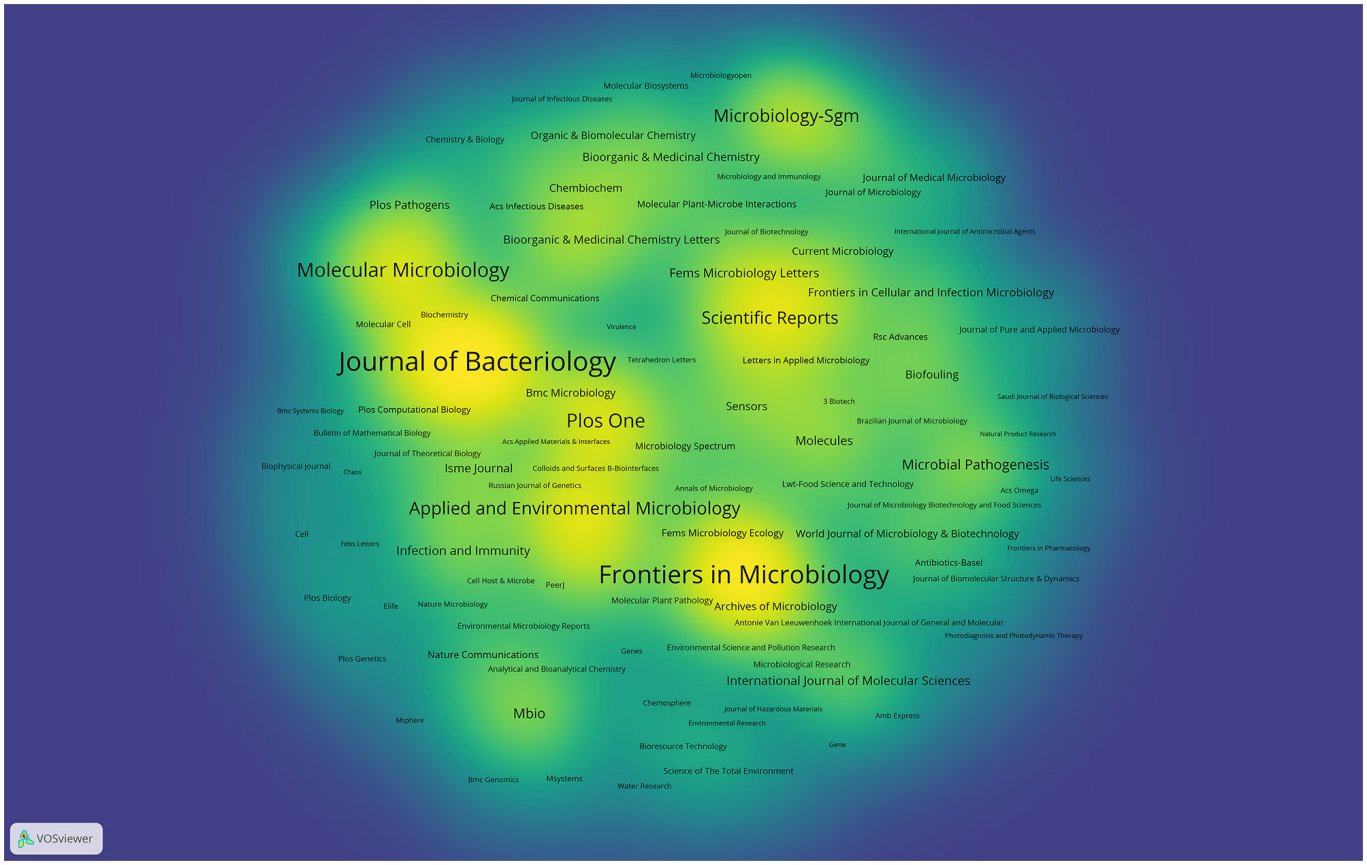
Density visualization map of journals on quorum sensing (2004–2023).

A total of 1,003 scholarly journals have made substantial contributions to disseminating QS -related research. Figure 7 provides an overview of journals excelling in this research domain. Among the 4,849 articles included in the QS database, 987 (comprising 20.35%) found their place in the top 10 journals (Table 2). Notably, Frontiers in Microbiology has the highest average citation number, closely followed by Journal of Bacteriology and PLoS ONE, collectively representing 9.3% of all articles in this field. Noteworthy among journals with more than 10 publications, Nature Microbiology (IF: 28.3), trailed by Nature Communications (IF: 16.6) and Nucleic Acids Research (IF: 14.9), all maintaining prestigious Q1 classification according to the JCR 2023 standard.

**Table 2:** Top 10 journals in the publication on quorum sensing (2004–2023).

**Rank**	**Journal**	**H-index**	**IF (2023)**	**Article counts**	**Percentage (*N* = 4,849)**	**Total number of citations**	**Average number of citations**
1	Frontiers in Microbiology	33	5.2	177	3.650%	3,916	22.12
2	Journal of Bacteriology	62	3.2	170	3.506%	11,737	69.04
3	PLoS ONE	38	3.7	104	2.145%	4,456	42.85
4	Molecular Microbiology	47	3.6	101	1.767%	7,258	71.86
5	Applied and Environmental Microbiology	38	4.4	82	1.691%	4,120	50.24
6	Scientific Reports	27	4.6	75	1.547%	2,232	29.76
7	Microbial Pathogenesis	27	3.8	74	1.526%	1,707	23.07
8	Microbiology SGM	39	2.8	74	1.526%	5,783	78.15
9	P Natl Acad Sci USA	47	11.1	69	1.423%	7,179	104.04
10	mBio	25	6.4	61	1.258%	1,903	31.2

### Authors' contributions to quorum sensing research

A total of 16,828 distinct authors made active contributions to this investigation, with Table 3 presenting the top 10 most prolific researchers. Noteworthy among them are Bassler, Bonnie L. from Princeton University and Howard Hughes Medical Institute, USA, renowned for her groundbreaking work in elucidating the molecular mechanisms of quorum sensing in bacteria; Williams, Paul from University of Nottingham, England, who has significantly advanced our understanding of bacterial biofilm formation and antibiotic resistance through his research on quorum sensing; and Blackwell, Helen E. from University of Wisconsin Madison, USA, acclaimed for her innovative synthesis of small molecules that modulate bacterial quorum sensing, all of whom stand as leading contributors in the field of microbiology and infectious disease control. Furthermore, we conducted an examination of highly cited articles within the QS research domain, with Table 4 showcasing the top 10 articles. Among these, the study titled “Quorum sensing: Cell-to-cell communication in bacteria” authored by Bassler et al., and published in Annual Review of Cell and Developmental Biology in 2005 (*n* = 2,676), emerges as the most frequently cited research (Table 4).

**Table 3:** The top 10 most productive authors contributed to publications in quorum sensing (2004–2023).

**Rank**	**Author**	**Article counts**	**H-index**	**Total number of citations**	**Average number of citations**
1	Bassler, Bonnie L.	91	50	14,955	164.34
2	Williams, Paul	75	42	6,580	87.73
3	Blackwell, Helen E.	73	34	3,232	44.27
4	Givskov, Michael	60	41	7,654	127.57
5	Greenberg, E. Peter	60	39	7,618	126.97
6	Chan, Kok-Gan	46	20	1,473	32.02
7	Venturi, Vittorio	39	24	1,826	46.82
8	Eberl, Leo	36	29	3,699	102.75
9	Zhang, Lian-Hui	34	21	2,456	72.24
10	Yin, Wai-Fong	30	17	1,121	37.37

**Table 4:** Top 10 cited articles on quorum sensing (2004–2023).

**Rank**	**Title**	**Journal**	**Corresponding author**	**Publication year**	**Total citations**
1	Quorum sensing: Cell-to-cell communication in bacteria	Annual Review of Cell and Developmental Biology	Bassler, Bonnie L.	2005	2,676
2	Quorum sensing signal-response systems in Gram-negative bacteria	Nature Reviews Microbiology	Bassler, Bonnie L.	2016	1,191
3	Bacterial Quorum-Sensing Network Architectures	Annual Review of Genetics	Bassler, Bonnie L.	2009	1,141
4	Bacterial Quorum Sensing: Its Role in Virulence and Possibilities for Its Control	Cold Spring Harbor Perspectives in Medicine	Bossier, Bonnie L.	2012	1,082
5	Sociomicrobiology: the connections between quorum sensing and biofilms	Trends in Microbiology	Greenberg, EP	2005	797
6	The small RNA chaperone Hfq and multiple small RNAs control quorum sensing in Vibrio harveyi and Vibrio cholerae	Cell	Bassler, Bonnie L.	2004	741
7	The hierarchy quorum sensing network in *Pseudomonas aeruginosa*	Protein & Cell	Zhang, Lianhui	2015	724
8	Progress in and promise of bacterial quorum sensing research	Nature	Greenberg, E. Peter	2017	671
9	Quorum Sensing in Staphylococci	Annual Review of Genetics	Geisinger, Edward	2008	649
10	Quorum sensing inhibitors: An overview	Biotechnology Advances	Kalia, Vipin Chandra	2013	591

### Research hotspots of quorum sensing

**Figure 8:**
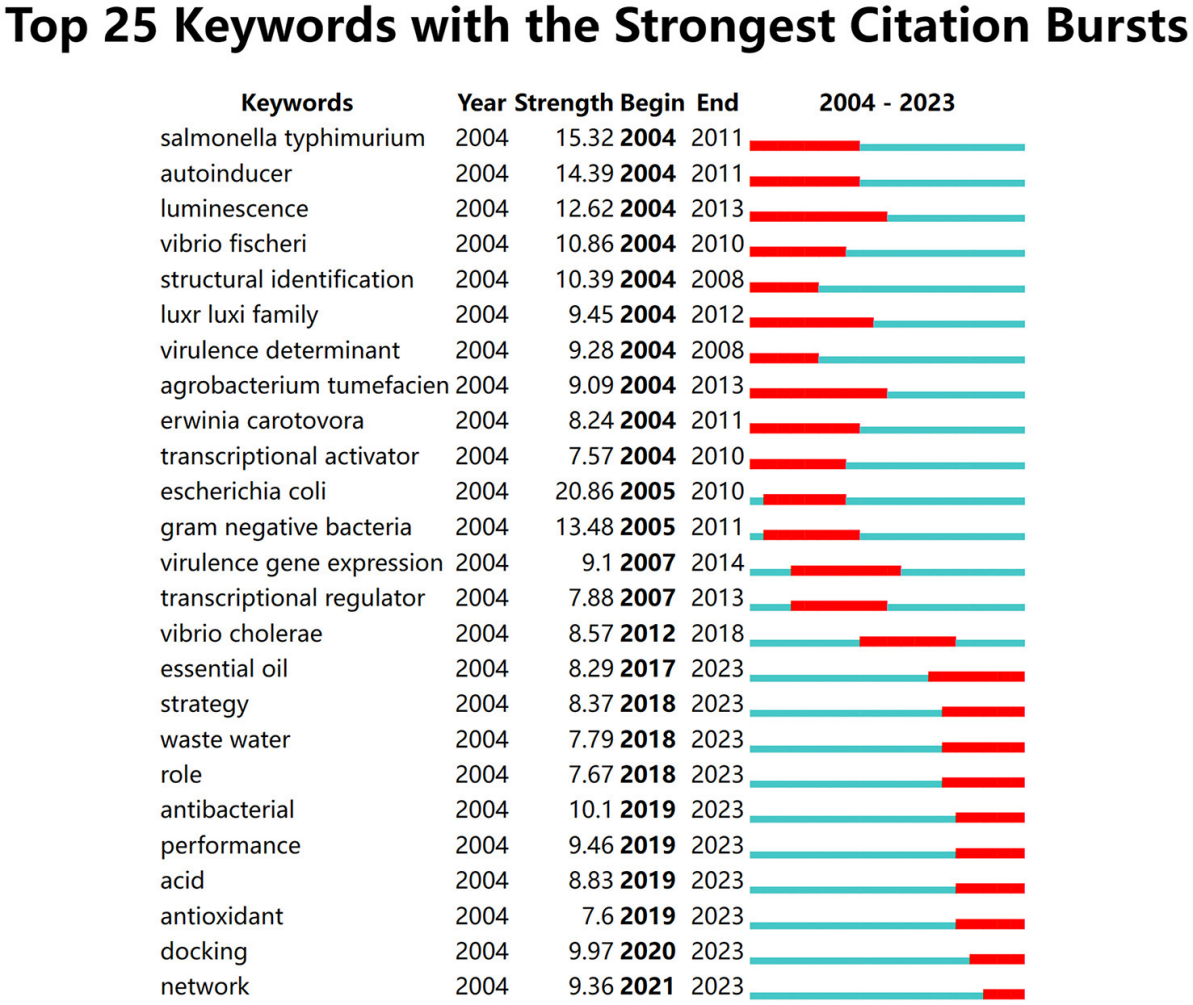
The top 25 burst words from 2004 to 2023.

We utilized CiteSpace for the extraction of keywords from a dataset encompassing 4,849 literature sources. This process led to the identification of the top 25 dynamic burst words, covering the period from 2004 to 2023, shedding light on the evolving trends in research hotspots (Figure 8). Furthermore, our analysis revealed a total of 2,707 significant MeSH terms/MeSH subheadings, with a collective frequency of 9,543 instances. High-frequency terms, defined according to the G-index criteria, were those recurring more than 31 times (Table 5).

**Table 5:** Major MeSH terms/MeSH subheadings from the included publications on quorum sensing (2004–2023).

**Rank**	**Major MeSH terms/MeSH subheadings**	**Frequency**	**Proportion of frequency (%)**	**Cumulative percentage (%)**
1	Quorum Sensing	790	8.2783	8.2783
2	Quorum Sensing/drug effects	531	5.5643	13.8426
3	Anti-Bacterial Agents/pharmacology	270	2.8293	16.6719
4	Quorum Sensing/physiology	263	2.7559	19.4279
5	Quorum Sensing/genetics	253	2.6512	22.0790
6	*Pseudomonas aeruginosa*/drug effects	205	2.1482	24.2272
7	Bacterial Proteins/metabolism	182	1.9072	26.1343
8	Biofilms/drug effects	162	1.6976	27.8319
9	*Pseudomonas aeruginosa*/physiology	126	1.3203	29.1523
10	Biofilms/growth and development	105	1.1003	30.2525
11	Gene Expression Regulation, Bacterial	102	1.0688	31.3214
12	Bacterial Proteins/genetics	100	1.0479	32.3693
13	*Pseudomonas aeruginosa*/metabolism	83	0.8697	33.2390
14	4-Butyrolactone/analogs and derivatives	82	0.8593	34.0983
15	*Pseudomonas aeruginosa*/genetics	67	0.7021	34.8004
16	Homoserine/analogs and derivatives	65	0.6811	35.4815
17	Acyl-Butyrolactones/metabolism	64	0.6706	36.1522
18	Plant Extracts/pharmacology	58	0.6078	36.7599
19	Trans-Activators/metabolism	58	0.6078	37.3677
20	Biofilms	52	0.5449	37.9126
21	Bacteria/metabolism	51	0.5344	38.4470
22	*Pseudomonas aeruginosa*	47	0.4925	38.9395
23	Bacterial Physiological Phenomena	43	0.4506	39.3901
24	Signal Transduction	43	0.4506	39.8407
25	Models, Biological	42	0.4401	40.2808
26	Chromobacterium/drug effects	41	0.4296	40.7105
27	Bacteria/drug effects	37	0.3877	41.0982
28	*Pseudomonas aeruginosa*/pathogenicity	37	0.3877	41.4859
29	Virulence Factors/metabolism	33	0.3458	41.8317
30	Lactones/metabolism	32	0.3353	42.1670
31	Virulence Factors/genetics	31	0.3248	42.4919

**Figure 9:**
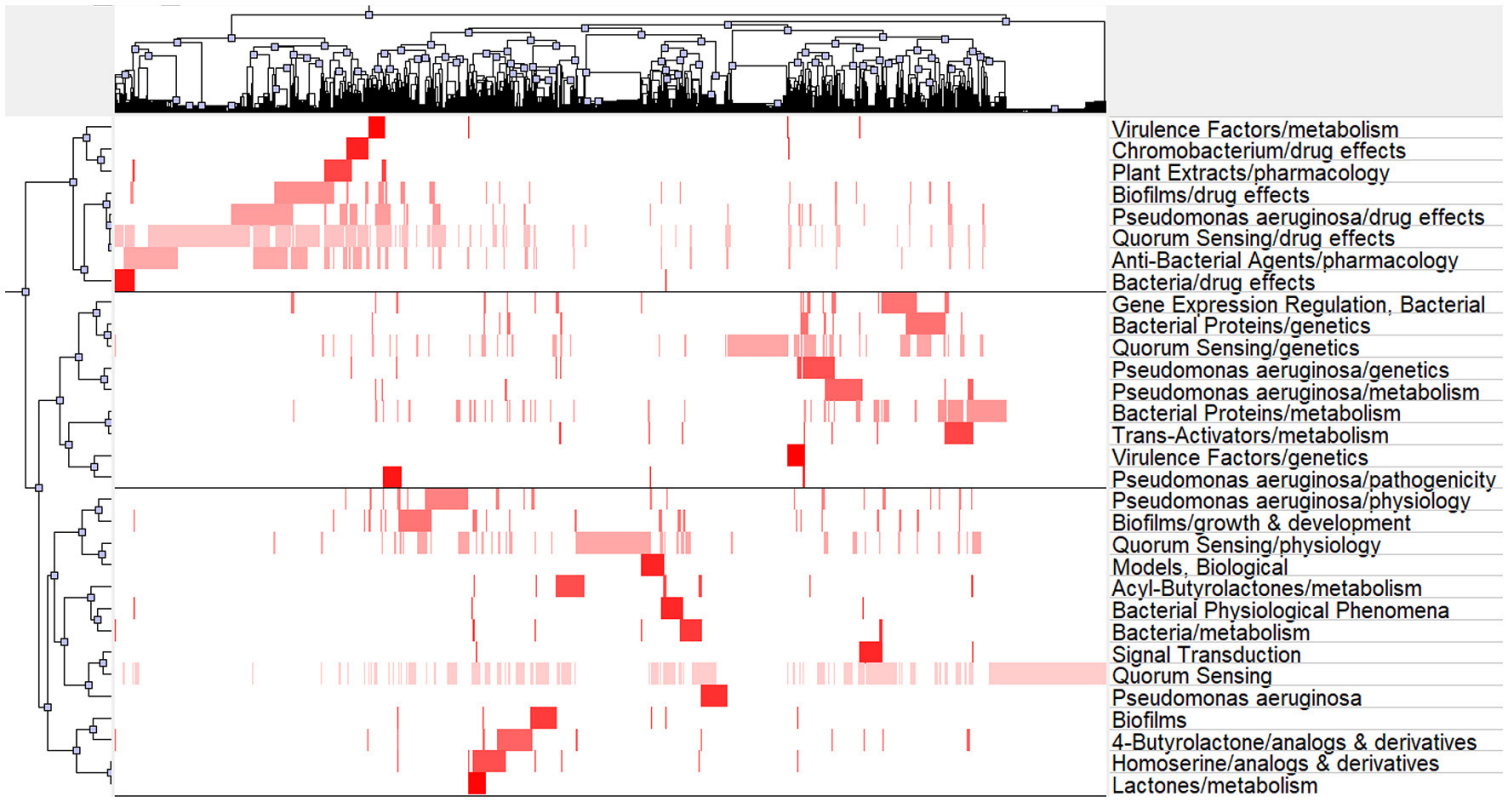
Matrix visualization of major MeSH terms/MeSH subheading terms of articles on quorum sensing.

**Figure 10:**
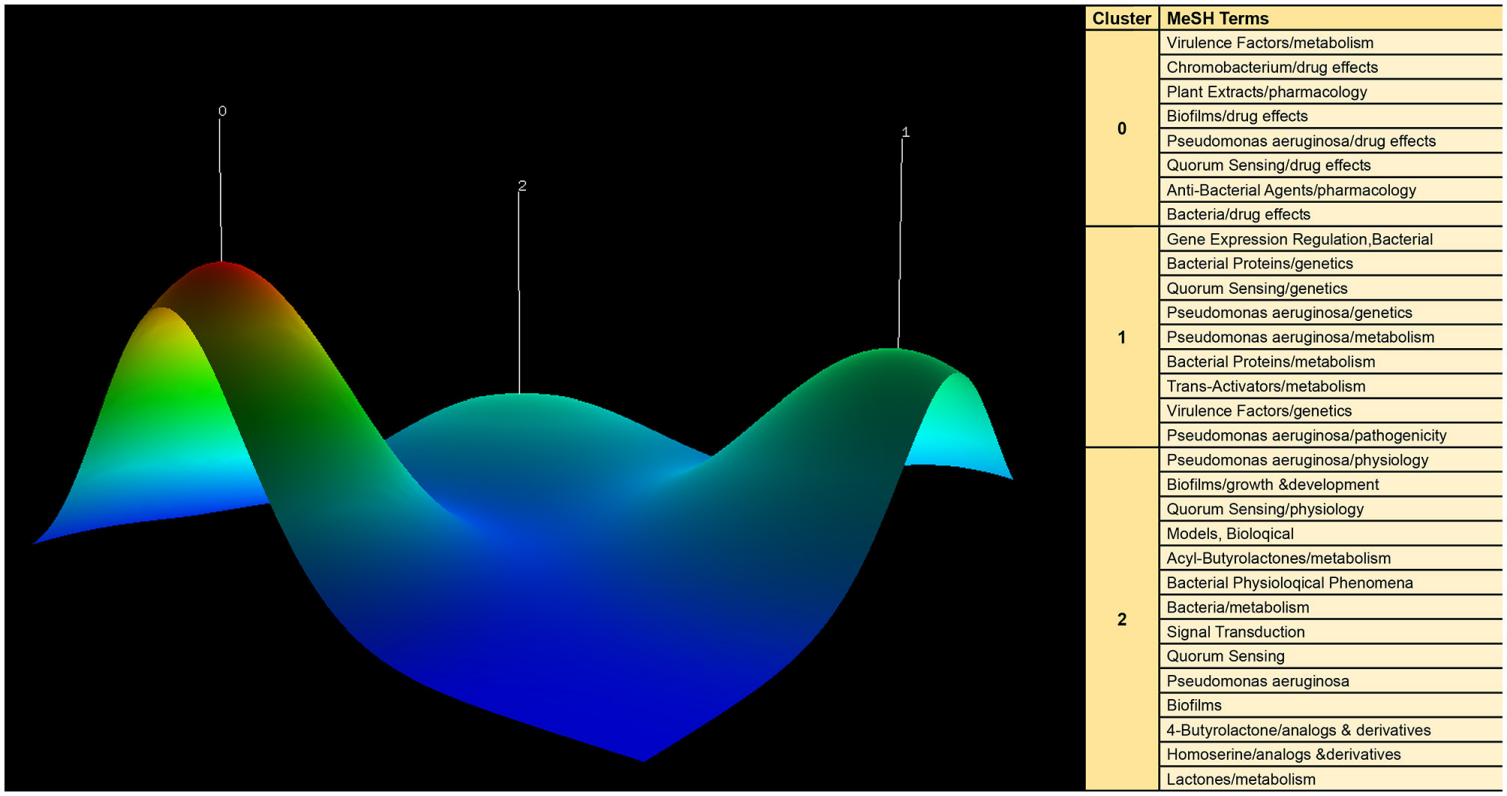
Mountain visualization of major MeSH terms/MeSH subheading terms of articles on quorum sensing.

In order to visualize these prominent research themes, we conducted biclustering analysis using BICOMB and gCLUTO. BICOMB was utilized to create a co-word matrix, which was then imported into gCLUTO to generate matrix visualization (Figure 9) and mountain visualization (Figure 10). The matrix on the left aligns with the primary MeSH terms and MeSH subheading terms depicted on the right. The visual distinction in the matrix blocks indicates the frequency of term occurrences. The mountain graph revealed the presence of three distinct clusters within the research domain. The gap between these peaks signifies the level of correlation between clusters, with mountain height and volume reflecting their internal similarity and term coverage. Additionally, the transition in colors, from red to green atop these peaks, corresponds to standard deviation levels. The biclustering analysis of the publications led to the identification of three primary clusters: (I) Drug effects and pharmacology of quorum sensing; (II) Genetics and metabolism of quorum sensing; (III) Physiology of quorum sensing.

## Discussion

The findings of the bibliometric analysis reveal a substantial expansion in the literature concerning QS from 2004 to 2023, signifying the increasing complexity of staying updated with current research trends. To address this challenge, we conducted a comprehensive investigation employing both bibliometric and biclustering analysis methodologies. This extensive study encompassed the retrieval of relevant articles from the WOS and NCBI databases, spanning two decades period. The outcomes of this inquiry are anticipated to yield valuable insights into research focal points and provide guidance on future trends.

### Overview of quorum sensing research

The exploration of QS mechanisms has unveiled a complex network of bacterial communication, surpassing the traditional boundaries of microbiological research and revealing its profound impact on microbiology, clinical medicine, and environmental science. For instance, the development of QS interference compounds could lead to significant advancements in preventing biofilm-associated infections. Moreover, the application of QS research in environmental engineering, especially in wastewater treatment, demonstrates tremendous potential, contributing to more efficient and environmentally friendly pollution control.

This study has evaluated national academic contributions and research quality within the QS domain, considering various factors such as article quantity, total citation count, centrality, and average citations per publication for each country or region. Notably, the results indicate that USA holds the most significant influence in QS research, accumulating a total of 1,292 article counts. In close pursuit, the China and India garnered 1,048 and 514, respectively. It's noteworthy that research on QS in the United States has exhibited a declining trend, while China has consistently increased its QS publications, surpassing the United States in 2018. China leads in influence (centrality = 0.32), ranking second among the top 10 countries. However, there is room for improvement in the quality of QS publications from China, as the average citations per study are relatively low (21.03), placing it last among the top 10 countries. This phenomenon may be associated with the impetuous atmosphere prevalent in the academic community, where there is a one-sided pursuit of the quantity of publications. The thermal world map highlights regions making substantial investments in QS research. The findings suggest that specific regions, such as Africa, Central Asia, and Eastern Europe, have limited participation in QS research and could enhance their contributions to this field through international collaboration and support.

The results reveal that nearly half of the top 10 institutions making substantial contributions to the field of QS are affiliated with the United States (*n* = 4), highlighting its significant impact. We utilized a visually engaging density visualization technique to represent distinct institution clusters, facilitating an intuitive evaluation of their collaborative networks. Institutions that collaborate closely are depicted within color-coded clusters, where the size of each cluster area signifies its importance in the field. Furthermore, the size of each institution's label corresponds to the extent of its collaboration with other research institutions. Prominent contributors to QS research include University of Nottingham (*n* = 129), Princeton University (*n* = 112), University of Wisconsin Madison (*n* = 90), Ghent University (*n* = 85).

The leading journals within the QS research domain, including Frontiers in Microbiology, Journal of Bacteriology, and PLoS ONE, have collectively made a substantial contribution, accounting for over 9% of the research output. Among these publications, Frontiers in Microbiology stands as the prominent leader, evident from its significantly higher article counts number of QS-related articles compared to its closest counterpart. Additionally, this journal boasts the high H-index, further underscoring its pivotal role in advancing knowledge within the QS field. These findings highlight the influential contributions of Frontiers in Microbiology in shaping the discourse and progression of QS research.

The examination of prominent terms in this field indicated a significant shift in the primary research focus between 2012 and 2018. Previously, emphasis centered primarily on the molecular mechanisms of QS, with a particular interest in bioactive compounds and their impact on bacterial cooperation, biofilm formation, and antibiotic resistance. In the contemporary landscape, the scope of research has expanded significantly to include investigations into novel QS inhibitors, antibacterial activities, and the use of advanced techniques like transcriptomics and synthetic biology to dissect the intricate workings of QS systems. This transformation underscores the dynamic nature of scientific exploration, as researchers adapt their inquiries to address pressing issues related to antimicrobial resistance, biofilm control, and the broader applications of QS research.

### Three clustering hotspots of quorum sensing research

#### Drug effects and pharmacology of quorum sensing

Cluster 0 presents a correlation with drug effects and pharmacology of QS. The research of QS has ushered in a new era of controlling bacterial behavior and physiological processes, highlighting the potential of drug effects and pharmacological interventions. Biofilms, complex communities of bacteria encased in a protective extracellular matrix, are notorious for their resistance to traditional antibiotics (Dufour et al., 2010). However, the development of anti-bacterial agents specifically targeting QS mechanisms has shown great promise in disrupting biofilm formation and rendering bacteria more susceptible to treatment (Bhardwaj et al., 2013; Zhao et al., 2013). Furthermore, QS inhibitors play a crucial role in mitigating toxin production. Many pathogens rely on QS to regulate the synthesis of virulence factors (Rutherford and Bassler, 2012). Inhibitors can significantly reduce the release of harmful substances by blocking these QS signals, thereby weakening the pathological effects caused by bacterial infections (Ni et al., 2009; El-Mowafy et al., 2014). QS inhibitors may play a key role in inhibiting the spread of antibiotic resistance genes by interfering with bacterial gene transfer mechanisms (Zhang et al., 2018). Additionally, they affect bacterial motility and iron acquisition, which are essential for bacterial colonization and the progression of infection (Sethupathy et al., 2016; Bahari et al., 2017). Currently, in the field of drug discovery, a variety of compounds, including furanone derivatives (Chang et al., 2019), benzimidazole derivatives (El-Gohary and Shaaban, 2017), Benzothiazole derivatives (Chu et al., 2023), Pyranone derivatives (Chen et al., 2019), Quinolines derivatives (Beus et al., 2020), Pyrrole derivatives (Hassan et al., 2016), Indole derivatives (Odularu et al., 2022), and others, have exhibited QS inhibitory properties.

It is noteworthy that various animal models are crucial for elucidating the therapeutic potential and mechanisms of these compounds. In addition to rats or mice, the use of *Caenorhabditis elegans* has provided a foundational understanding of the effects of QS inhibitors on host-pathogen interactions (Husain et al., 2016; Liu et al., 2020). Similarly, the zebrafish model, owing to its transparent embryonic development, has become a valuable tool for the visualization of microbial infections *in vivo* (Papaioannou et al., 2013; Nogaret et al., 2021). These models collectively contribute to the advancement of research on QS inhibitors. Thus, it is essential to emphasize the importance of these animal models in propelling the research on QS inhibitors forward. And antibodies designed to quench QS signaling represent a biotechnological approach that has gained traction in recent years. By specifically targeting QS molecules, these antibodies disrupt bacterial communication pathways and interfere with the expression of virulence factors and biofilm formation (Ahmed et al., 2019; Munir et al., 2020; Zhou et al., 2020; Gunaratnam et al., 2021). Moreover, the utilization of biotic polymers and polymeric materials in QS inhibition is a promising approach (Lu et al., 2022; Tajani et al., 2022). These materials are engineered to deter bacterial adhesion and biofilm development on surfaces, making them invaluable for reducing the risk of infections associated with medical devices and industrial settings.

In summary, in seeking innovative antibacterial strategies, QS inhibitors differ from traditional antibiotics in their bactericidal or bacteriostatic effects, reducing the selective pressure on bacteria, which, in theory, could lower bacterial resistance. Moreover, QS inhibitors exemplify a targeted antimicrobial strategy that can minimize harm to beneficial microbial communities, thus potentially reducing adverse effects associated with antibiotic treatment (Delago et al., 2016; Kang et al., 2019). Integrating QS inhibitors into treatment regimens could enhance pathogens' sensitivity to existing drugs, thereby improving the efficacy of antibiotics (Brackman et al., 2011; Sun et al., 2019). However, the complexity of translating laboratory findings into clinical applications must be acknowledged. Despite the proven effectiveness of various QS inhibitors *in vitro*, their transition to clinical application faces several challenges. Firstly, bacterial QS systems are diverse and complex, with different species using different signaling molecules and receptors, making the development of broad-spectrum QS inhibitors or those targeting specific bacterial QS systems difficult and limited. Secondly, in the complex environment of the human body, factors like pH and enzyme activity can affect their functionality, potentially impacting the efficiency and stability of QS inhibitors. Most importantly, the safety and specificity of these inhibitors require rigorous toxicity assessments and multi-phase clinical trials. Therefore, although QS inhibitors are theoretically a promising antimicrobial strategy, overcoming these challenges and successfully applying them to clinical practice still requires further research and development.

#### Genetics and metabolism of quorum sensing

Cluster 1 indicates the correlations with genetics and metabolism of QS. QS plays a pivotal role in orchestrating cooperative gene expression and metabolic adaptations within bacterial populations. This intricate genetic and metabolic regulation enables bacteria to synchronize their behavior, fostering cooperation within the population while enhancing competitiveness among different bacterial communities (Ng and Bassler, 2009; Schluter et al., 2016; Abisado et al., 2018). When the QS pathway is upregulated in pathogens, the burden on pathogen metabolism primarily manifests in the allocation of energy and resources. To respond to QS signaling molecules, pathogens need to invest substantial metabolic resources, which include but are not limited to ATP and precursor molecules (Vendeville et al., 2005; Goo et al., 2015). This reallocation of resources may result in a weakening of other physiological functions in the pathogen, such as a potential slowdown in growth rate (Albuquerque et al., 2014). Although the upregulation of the QS pathway can increase the pathogenicity of the pathogen, it also places stress on its metabolism.

One of the key features of QS is its ability to coordinate gene expression across multiple bacterial cells, leading to the emergence of collective group behaviors (Antonioli et al., 2019; Striednig and Hilbi, 2022). These behaviors encompass a wide range of vital processes, such as biofilm formation, virulence factor expression, and swarming motility (Castillo et al., 2014; Li et al., 2018). Such coordinated alterations in gene expression are central to understanding the physiological and ecological significance of QS in bacterial communities. Autoinducer-dependent gene expression regulation is a hallmark of QS, where signaling molecules, such as AHL, mediate the communication between bacteria (Czajkowski and Jafra, 2009; LaRock et al., 2013; Calatrava-Morales et al., 2018). AHL-mediated QS is intricately linked to substance metabolism and energy utilization. For instance, in the opportunistic pathogen Pseudomonas aeruginosa, AHL serves as the principal signaling molecule controlling the expression of ~300 genes involved in various cellular functions, including pathogenesis (Brindhadevi et al., 2020). This molecular dialogue, facilitated by AHL, underscores the profound impact of QS on bacterial metabolism, influencing the utilization of resources and the expression of virulence factors essential for host colonization and infection (Haque et al., 2018; Warrier et al., 2021; Zhu et al., 2023).

In some species, there are multiple QS pathways, aimed at providing more complex and nuanced behavioral regulation. This intricate regulatory system allows bacteria to respond to a variety of internal and external signals under different environmental conditions, implementing more flexible and efficient survival strategies. Each QS system can specifically sense different signal molecules and trigger specific gene expression patterns, enabling bacteria to adjust their metabolic pathways, production of virulence factors, and biofilm formation in response to changes in environmental conditions and population density (Miller and Bassler, 2001). Moreover, cross-communication between multiple QS pathways is very common, which can either promote or inhibit each other. Furthermore, QS's role extends beyond mere bacterial communication, acting as a pivotal driver in the evolutionary and adaptive processes of microbial life. Its integration with mobile genetic elements, such as conjugative elements (Qixin et al., 2022), fosters horizontal gene transfer among diverse bacterial species (Brameyer and Heermann, 2015; Hoover et al., 2015). Through this mechanism, bacteria are able to integrate information from different signals, allowing them to coordinate their behavior in specific environments and enhance their adaptability.

By delving into the genetic and metabolic frameworks underpinning QS, researchers unravel the intricate mechanisms of bacterial behaviors. This knowledge not only sheds light on the molecular underpinnings of microbial consortia but also reveals the profound influence of QS on microbial evolution, community structuring, and ecological function. Therefore, exploring the depths of QS systems offers valuable insights into microbial ecology, promising advancements in biotechnological applications and novel approaches in combating bacterial pathogenicity.

#### Physiology of quorum sensing

Cluster 2 presents the correlations with physiology of QS. In these complex ecosystems, various bacterial species utilize specific QS signal molecules to establish communication, enabling them to engage in mutually beneficial symbiotic relationships or to compete for essential resources (Hoover et al., 2015; Qixin et al., 2022). This intricate web of interspecies interactions is facilitated by a diverse array of QS signal molecules, including AHLs (Brameyer and Heermann, 2015), autoinducer-2 (AI-2; Hooshangi and Bentley, 2008; Zhao et al., 2018), extracellular death factor (EDF; León-Félix and Villicaña, 2021), γ-butyrolactone (GBL; Decho et al., 2010), diffusible signal factor (DSF; He et al., 2023), and α-hydroxyketones (AHKs; Mangwani et al., 2016). Notably, AHKs and DSF have been shown to mediate signal communication not only between bacterial species but also between fungi and even between eukaryotic host cells (Ahn et al., 2022; Fan et al., 2022). In addition, nitric oxide is involved in a variety of physiological processes, including antimicrobial activities in immune responses (MacMicking et al., 1997). At the same time, the presence of nitric oxide can also be sensed by bacteria through their QS system, thereby regulating their metabolic behavior in response to these signals. For example, nitric oxide (NO) in influencing the autophosphorylation of partner histidine kinases integrated in the QS pathway of *Vibrio* (Heckler and Boon, 2019). The existence of these signals reveals the complex interactions between hosts and microbes.

Advancements in QS research have revealed the specificity of signal transduction mechanisms across different species. Particularly, the LuxI-LuxR pathway plays a crucial role in the regulation of virulence, biofilm formation, and antibiotic resistance in various Gram-negative bacteria. For instance, *P. aeruginosa* regulates virulence genes through QS circuits, including the LasI/LasR and RhlI/RhlR systems. These systems control the production of virulence factors essential for the bacterium's infection capability and survival under diverse environmental conditions (Banerjee and Ray, 2016). On the other hand, Gram-positive bacteria primarily utilize autoinducing peptides (AIPs). The system in *S. pneumoniae* and *B. subtilis* exemplifies a QS pathway where a competence stimulating peptide activates receptor kinase to trigger DNA uptake, showcasing QS's role in competence (Miller and Bassler, 2001; Wolf et al., 2016). Similarly, the accessory gene regulator (Agr) system in *S. aureus* and *C. perfringens* underlines QS's importance in virulence factor regulation (Wang and Muir, 2016), with the Agr locus orchestrating a phosphorylation relay that upregulates Agr genes and virulence factors like δ-toxin (Bhatt, 2018).

### Limitation

The utilization of bibliometric analysis in research is not without limitations. Among these constraints is the potential omission of high-quality research from the dataset due to inadequate citation frequency. This situation may arise because recently published articles might not have had ample time to accumulate citations, which constitutes a significant basis for bibliometric assessment (Wallin, 2005). Additionally, the biclustering analysis employed for categorizing and identifying pertinent keywords may not encompass all relevant subjects within a research field, such as this study primarily focusing on the biomedical field. Furthermore, data derived from the Web of Science database might not encompass the most recent publications owing to update delays. Nonetheless, it is imperative to acknowledge that bibliometric analysis retains its value as a tool for achieving a comprehensive and meaningful comprehension of the research landscape within a specific domain.

## Conclusions

In conclusion, this bibliometric analysis has illuminated significant aspects of QS research, shedding light on its evolving trajectory and its growing importance within the scientific community. Our examination of QS literature has revealed a consistent upward trend in research output, reflecting the expanding interest and recognition of QS as a pivotal mechanism in microbial communication and its implications across various disciplines. By identifying key contributors, prolific journals, and prominent geographical regions, this study provides critical insights into the landscape of QS research. Moreover, the diversified applications of QS in biotechnology, medicine, and ecology underscore its multifaceted significance. Therefore, collaboration among microbiologists, medical scientists, ecologists, computer scientists, and others is encouraged. Interdisciplinary research can facilitate breakthroughs in understanding the mechanisms of QS and its applications in biotechnology, medicine, and environmental science. As we delve deeper into the intricate web of QS, it becomes evident that this field's potential remains largely untapped, with ample opportunities for future exploration and innovation. The physiology, pharmacology, genetics, and metabolism of QS are research hotspots in the biomedical field. Moreover, the importance of researching novel QS inhibitors is increasingly emphasized. Through a comprehensive analysis of QS research hotspots, we have identified the tremendous potential of QS research for developing new therapeutic strategies and eco-friendly biotechnological applications. Furthermore, we discuss the importance of transforming research outcomes of QS inhibitors into practical applications. These findings not only consolidate the current state of QS research but also provide a valuable foundation for guiding future endeavors in unraveling the complexities of microbial communication and its wide-ranging impacts. Anticipatedly, this study aims to offer valuable support and direction to scholars and practitioners engaged in this particular domain.

## Data availability statement

The original contributions presented in the study are included in the article/supplementary material, further inquiries can be directed to the corresponding authors.

## Author contributions

XC: Conceptualization, Formal analysis, Methodology, Software, Supervision, Writing – original draft, Visualization. JL: Conceptualization, Formal analysis, Software, Writing – original draft, Visualization. RL: Data curation, Formal analysis, Software, Writing – original draft, Supervision. XS: Data curation, Writing – original draft, Software. YX: Data curation, Software, Writing – original draft. XX: Writing – original draft, Conceptualization, Supervision. HX: Conceptualization, Funding acquisition, Writing – review & editing, Supervision. DX: Conceptualization, Funding acquisition, Project administration, Visualization, Writing – review & editing.
